# The effects of childhood maltreatment on adolescent non-suicidal self-injury behavior: mediating role of impulsivity

**DOI:** 10.3389/fpsyt.2023.1139705

**Published:** 2023-05-26

**Authors:** Xi Li, Xiao-Li Liu, Yu-Jing Wang, Dong-Sheng Zhou, Ti-Fei Yuan

**Affiliations:** ^1^School of Mental Health, Wenzhou Medical University, Wenzhou, China; ^2^Department of Psychiatry, Ningbo Kangning Hospital, Ningbo, China; ^3^Shanghai Key Laboratory of Psychotic Disorders, Shanghai Mental Health Center, Shanghai Jiao Tong University School of Medicine, Shanghai, China; ^4^Co-innovation Center of Neuroregeneration, Nantong University, Nantong, China

**Keywords:** childhood maltreatment, emotional abuse, impulsivity, non-suicidal self-injury, adolescent

## Abstract

**Background:**

Non-suicidal self-injury (NSSI) severely challenges mental health in adolescents. Childhood maltreatment experience acts as high-risk factor for adolescents to engage in NSSI behaviors. On the other hand, impulsivity or loss of control sets the threshold for NSSI execution. Here we examined the effects of childhood maltreatment on adolescent NSSI-related clinical outcomes and the potential role of impulsivity.

**Methods:**

We assessed the clinical data of 160 hospitalized NSSI adolescents and recruited 64 age-matched healthy subjects as a control group. The clinical symptoms of NSSI are expressed by the NSSI frequency, depression, and anxiety measured by the Ottawa Self-Injury Inventory, the Beck Depression Inventory, and the Beck Anxiety Inventory. Childhood maltreatment and impulsivity were assessed with Childhood Trauma Questionnaire and Barratt Impulsiveness Scale.

**Results:**

The results showed that when compared to HC group, NSSI group is more likely to experience childhood maltreatment. Notably, NSSI group with Childhood maltreatment accompanies higher trait impulsivity and exacerbated clinical outcomes, such as NSSI frequency, depression and anxiety symptoms. Mediation analyses indicated that the association between childhood maltreatment and NSSI-related clinical outcomes was partially explained by impulsivity.

**Conclusion:**

We found that NSSI adolescents have a higher proportion of childhood maltreatment. Impulsivity plays a mediating role between childhood maltreatment and NSSI behaviors.

## Introduction

Non-suicidal self-injury (NSSI) is the direct and intentional destruction of body tissue without any intent to die (1). Adolescents are at high risk of developing NSSI behaviors. The prevalence of NSSI in children and adolescents is about 19.5% worldwide and about 27.4% in the Chinese population aged 13 to 18 years (2, 3). NSSI commonly aims to reduce negative emotions, such as stress, anxiety, and self-blame (4–7). Individuals frequently report experiencing an immediate sense of relief during self-injury (8). In addition to being a severe threat to adolescents’ mental and physical health, NSSI can also cause severe social impacts and economic burdens (9, 10). Understanding the risk factors and early prevention is critical in reducing the harm caused by NSSI.

Childhood maltreatment could increase the risk of experiencing mental disorders in adolescence and adulthood (11–13). Childhood maltreatment typically includes five subtypes: emotional abuse, physical abuse, sexual abuse, emotional neglect, and physical neglect. Previous studies indicate that childhood maltreatment is a risk factor for developing NSSI behaviors, and any type of childhood maltreatment increases an individual’s risk of engaging in NSSI behaviors (14, 15). In addition, some studies have found that individuals with Childhood maltreatment report more severe depression and anxiety symptoms (16, 17). Childhood maltreatment is associated with symptoms of depression and anxiety, including in NSSI adolescents (18). A retrospective study of a large sample reported that participants who were abused during childhood were more likely to have more psychiatric problems at age 18, including depression, anxiety, self-injury, and alcohol dependence (19). The abuse experience was also associated with poorer treatment outcomes, with a one-year follow-up of outpatients found that depressed patients with a history of maltreatment were more difficult to remit (20). Given childhood maltreatment’s devastating and long-term negative effects, the mechanisms underlying the complex relationship between traumatic childhood experiences and NSSI behaviors have also attracted increasing attention.

One possible factor of childhood maltreatment influencing NSSI behavior is impulsivity (21). Impulsivity is a complex concept involving the pathophysiology of multiple psychiatric disorders (22). In both the community and hospital samples, NSSI adolescents reported stronger impulsivity (23, 24). Impulsivity was associated with more severe depression and anxiety symptoms (25). It is well known that both childhood maltreatment and high impulsivity are important risk factors for NSSI, but few studies have explored whether they interact with NSSI. Individual studies have focused on one type of childhood maltreatment. A meta-analysis found that childhood maltreatment was positively correlated with trait impulsivity, particularly in emotional abuse (21). Another study of adults experiencing sexual abuse also found that, impulsivity explained 11% of the association between suicidal self-injury and sexual abuse, and 4% of the association between NSSI and sexual abuse (26). More suicide-related studies revealed that childhood maltreatment could directly predict suicidal behavior, and impulsivity partially mediates the pathway (27–29). These evidences suggest that childhood maltreatment and impulsivity are indeed correlated in suicidal populations and work together on suicidal behavior. Since NSSI partially overlaps with the neurobiological characteristics of suicidal behavior, and we speculate that impulsivity may also mediate the effects of childhood maltreatment on NSSI-related clinical outcomes. At present, few studies have comprehensively explored the role of impulsivity in the association between childhood maltreatment and multiple clinical outcomes in NSSI adolescents, but the implementation of such research is conducive to the prevention of NSSI.

**Figure 1:**
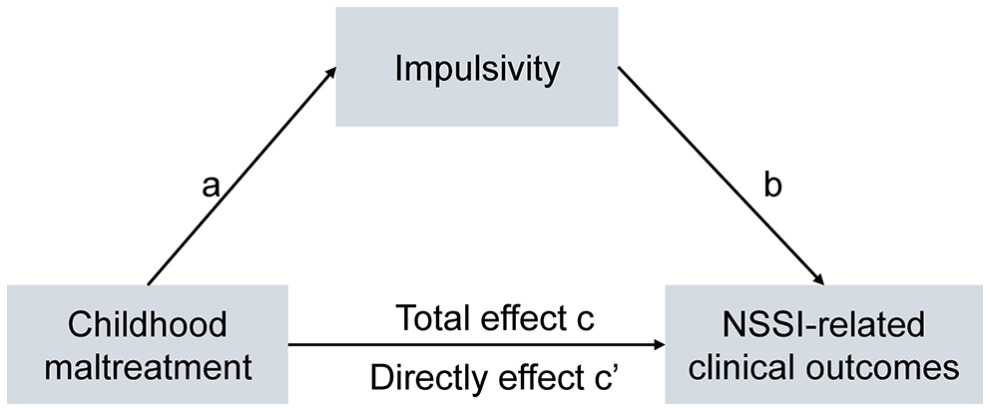
The theoretical model.

Here, considering that childhood maltreatment and high impulsivity are associated with more severe clinical outcomes in adolescents with NSSI, we aimed to (1) characterize the performance of childhood maltreatment and impulsivity in NSSI adolescents, (2) examine the effects of having childhood maltreatment on impulsivity and NSSI behavior. Based on previous findings of the relationship between childhood maltreatment, impulsivity, and more severe clinical symptoms (more frequent NSSI, anxiety, depression), We further validated a theoretical mediation model of whether impulsivity mediates the relationship between childhood maltreatment and NSSI-related clinical symptoms (Figure 1).

## Materials and methods

### Participants

A total of 160 NSSI adolescent inpatients of Ningbo Kangning Hospital and 64 healthy controls (HC) were recruited from April 2021 to July 2022. Inclusion criteria for NSSI adolescents were as follows: (1) age between 12 and 18 years; (2) DSM-5 diagnosis of major depressive disorders, anxiety disorders, and bipolar I disorder (major depressive episode); and (3) five or more days engaged in NSSI during the past year. Exclusion criteria were (1) history of modified electroconvulsive therapy (MECT) treatment within the past 6 months; (2) suicide attempts during the last year; (3) suicidal plans or acute risk for suicide; (4) unable to understand or comply with the study procedure. All HC adolescents had no history of psychiatric disorders and self-injury. Two professional psychiatrists completed the entire interview process for the entry screening. All adolescent participants and their legal guardians signed written informed consent, and the study was approved by the ethics committee from Ningbo Kangning Hospital.

### Demographics and clinical characteristics

Demographic and clinical information, including sex, age, grade, only one-child family, urbanization and family history of mental disorders, were collected from all participants. The Beck Anxiety Inventory (BAI) was used to access current self-reported levels of anxiety. Twenty-one items were scored using a 4-point Likert scale (0–3). Higher scores indicate higher levels of anxiety. To access the severity of depressive symptoms, we used the Beck Depression Inventory (BDI), a four-point (0–3), 21-item self-assessment scale. In this sample, Cronbach’s alpha of BAI and BDI are 0.938 and 0.947, respectively. For NSSI adolescents, NSSI-related information was accessed using the Ottawa Self-Injury Inventory (OSI) (30). NSSI frequency in the past month was measured by the question “How often in the past month have you actually injured yourself, without the intention to kill yourself?” options are “not at all,” “at least once,” “weekly,” and “daily,” with a score of 0–3.

### Childhood trauma questionnaire

Childhood maltreatment experiences were assessed using the Childhood Trauma Questionnaire (CTQ) (31). This 28-item self-report scale is scored on a five-point Likert scale ranging from 1 (never true) to 5 (very often true), consisting of 5 subscales: emotional abuse, physical abuse, sexual abuse, emotional neglect, and physical neglect. Physical abuse ≥10, sexual abuse ≥8, emotional abuse ≥13, physical neglect ≥10, and emotional neglect ≥15 was considered trauma exposure (32). Cronbach’s alpha for CTQ in our sample was 0.812.

### Barratt impulsiveness scale (BIS-11)

Trait impulsivity was assessed by the 30-item Barratt Impulsiveness Scale (BIS-11), which includes three dimensions: nonplanning impulsivity, motor impulsivity, and attentional impulsivity. Each subscale contains 10 items with a five-point Likert scale (1 “never” to 5 “very often”), with a higher score indicating stronger trait impulsivity. The Chinese version of BIS-11 used in this study has good reliability and validity (33). The Cronbach’s alpha for BIS was 0.916 in the present study.

### Statistical analysis

Comparisons of the clinical and demographic variables between HC and NSSI groups were performed by independent *t*-tests for continuous variables and chi-square test for categorical variables. Mann–Whitney U tests were used to test for differences in impulsivity and clinical outcomes between those with and without childhood maltreatment within the NSSI group. Spearman correlation was used to explore the relationship between different types of childhood maltreatment, impulsivity, and clinical outcomes in NSSI group. To verify the mediation pathway from childhood maltreatment, through the role of impulsivity, to clinical outcomes of NSSI, we further performed three independent mediation effect testing. Harman’s single-factor test (34) was used to test for possible common method bias. The mediation effects were carried out using the PROCESS macro (35) for SPSS (version 26, IBM), and 5,000 bootstrap samples were constructed for the significance of mediation effects. If the 95% confidence interval of the path coefficient does not contain 0, the effect would be significant.

## Results

### Group differences in demographic and clinical characteristics

The basic characteristics of all participants are shown in Table 1. There were no significant differences between HC and NSSI groups for age, education years, sex, only one-child family, urbanization and family history of mental disorders (all *p* > 0.05). The mean (SD) score of the NSSI frequency (past month) was 1.76 (0.95), and the onset age of NSSI was 12.62 (1.663). NSSI adolescents have more severe depressive (*t* = −17.071, *p* < 0.001, Cohen’s *d* = −2.112) and anxiety symptoms (*t* = −15.701, *p* < 0.001, Cohen’s *d* = −1.914) symptoms than HC adolescents. The level of BIS total (*t* = −7.205, *p* < 0.001, Cohen’s *d* = −1.064) was significantly higher in NSSI adolescents compared to HC. Moreover, NSSI adolescents also experienced more childhood maltreatment, with higher scores in emotional abuse (*t* = −9.972, *p* < 0.001, Cohen’s *d =* −1.300), physical abuse (*t* = −7.342, *p* < 0.001, Cohen’s *d* = −0.883), sexual abuse (*t* = −5.000, *p* < 0.001, Cohen’s *d =* −0.565), emotional neglect (*t* = −9.250, *p* < 0.001, Cohen’s *d =* −1.307), physical neglect (*t* = −5.510, *p* < 0.001, Cohen’s *d =* −0.829), and CTQ total (*t* = −11.605, *p* < 0.001, Cohen’s *d =* −1.539) than HC adolescents (Table 1).

**Table 1:** Demographics and clinical characteristics in the study population.

	HC (*N* = 64)	NSSI (*N* = 160)	Statistic (*χ*^2^/*t*)	*p*	Cohen’s *d*
Demographic					
Age, year	14.25(1.48)	14.16(1.396)	0.417	0.677	–
Education, year	8.36(1.418)	8.38(1.4)	−0.075	0.94	–
Sex (M, F)	12,52	26,134	0.203	0.652	–
Only one-child family (Yes, No)	25,39	73,87	0.8	0.371	–
Urbanization (Urban, Rural)	44,20	94,66	1.933	0.164	–
Primary diagnosis, No.			–	–	–
Depression	–	120			
Anxiety	–	35			
Bipolar I disorder (Major depressive episode)	–	5			
Clinical characteristics					
Family history of mental disorders (Yes, No)	0,64	7,153	2.89	0.089	–
NSSI frequency score (past month)	–	1.76(0.95)	–	–	–
Onset age	–	12.62(1.663)	–	–	–
BAI	4.52(5.246)	24.53(13.822)	−15.701	< 0.001	−1.914
BDI	5.63(6.572)	30.94(15.62)	−17.071	< 0.001	−2.112
BIS total	39.075(16.540)	56.599(16.405)	−7.205	< 0.001	−1.064
CTQ total	36.16(8.985)	54.94(14.739)	−11.605	< 0.001	−1.539
Emotional abuse	6.91(3.074)	12.68(5.47)	−9.972	< 0.001	−1.300
Physical abuse	5.56(1.489)	8.48(4.431)	−7.342	< 0.001	−0.883
Sexual abuse	5.08(0.37)	6.39(3.26)	−5.000	< 0.001	−0.565
Emotional neglect	10.94(3.984)	16.77(4.89)	−9.250	< 0.001	−1.307
Physical neglect	7.67(3.432)	10.63(3.707)	−5.510	< 0.001	−0.829

BAI, beck anxiety inventory; BDI, beck depression inventory; BIS, barratt impulsiveness scale; CTQ, childhood trauma questionnaire; HC, healthy controls adolescents; NSSI, non-suicidal self-injury adolescents.

### Differences in clinical characteristics of NSSI adolescents with and without experience of childhood maltreatment

**Figure 2:**
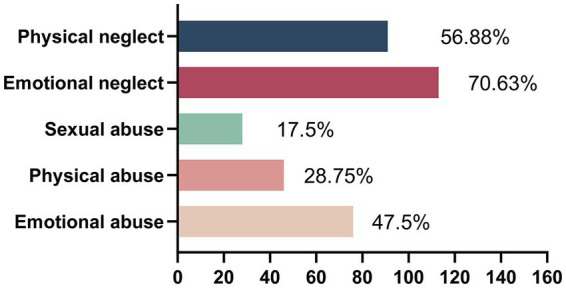
The prevalence of child maltreatment subtypes in NSSI group.

**Figure 3:**
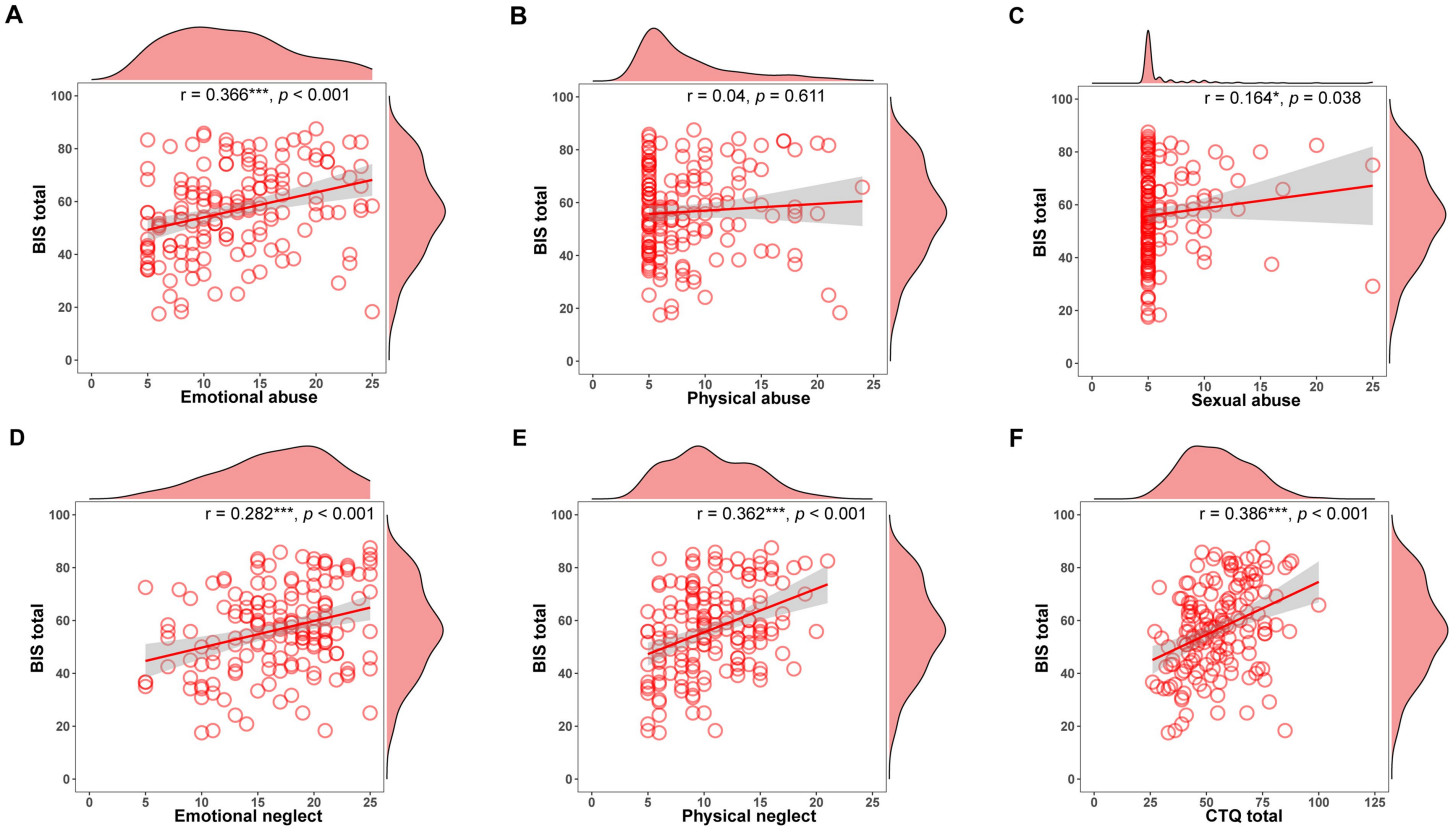
The relationship between childhood maltreatment subtypes and impulsivity in NSSI group. **(A)** Correlation between BIS total and emotional abuse. **(B)** Correlation between BIS total and physical abuse. **(C)** Correlation between BIS total and sexual abuse. **(D)** Correlation between BIS total and emotional neglect. **(E)** Correlation between BIS total and physical neglect. **(F)** Correlation between BIS total and CTQ total. BIS, barratt impulsivity scale; CTQ, childhood trauma questionnaire. *p < 0.05; **p < 0.01; ***p < 0.001.

In our NSSI hospitalized adolescents, 47.5% experienced emotional abuse, 28.75% experienced physical abuse, 17.5% experienced sexual abuse, and 70.63 and 56.88% reported emotional neglect and physical neglect (Figure 2). NSSI adolescents who have experienced emotional abuse have more frequency of self-injury (Supplementary Table S1). NSSI adolescents with emotional abuse, sexual abuse, emotional neglect, and physical neglect have more symptoms of depression and anxiety (Supplementary Tables S1–S5). Figure 3 presents the relationship between childhood maltreatment subtypes and impulsivity. Emotional abuse (*r* = 0.366, *p* < 0.001), emotional neglect (*r* = 0.282, *p* < 0.001), physical neglect (*r* = 0.362, *p* < 0.001) and CTQ total score (*r* = 0.386, *p* < 0.001) were positively correlated with BIS total score. The sexual abuse score was marginally correlated with BIS total score (*r* = 0.164, *p* = 0.038).

### Mediation effect

The association between childhood maltreatment, impulsivity, and clinical outcome was evaluated (Supplementary Table S6). Emotional abuse (*r* = 0.245, *p* = 0.002) and BIS total score (*r* = 0.319, *p* < 0.001) were positively correlated with NSSI frequency scores (past month). CTQ total and BIS total were significantly associated with BDI and BAI. Consequently, further mediation models were constructed separately for childhood maltreatment, impulsivity, and NSSI clinical outcomes.

After controlling for sex and age, the mediating effects of impulsivity between emotional abuse and NSSI frequency scores (past month) were tested. Impulsivity mediated 31.66% of the emotional abuse on NSSI frequency scores (past month). In addition, two separate mediation analyses examined the indirect effect of childhood maltreatment total on BDI and BAI, respectively, which was mediated by impulsivity. Impulsivity mediated 40.10% of the effect of childhood maltreatment total on BDI and 25.09% of the effect on BAI. Table 2 shows the results of the mediation analysis.

**Table 2:** Impulsivity as a mediator in the relationship between childhood maltreatment and clinical outcome (total effect, direct effect, and indirect effect).

	Total effect, estimate (S.E.), *p* value	Direct effect, estimate (S.E.), *p* value	Indirect effect, bootstrap confidence interval	Partially standardized indirect effect	Proportion mediated %
NSSI frequency score (past month)	0.0398, (0.0134) *p* = 0.003	0.0272, (0.0136) *p* = 0.047	0.0042–0.0233	0.0132	31.66%
BDI	0.4374, (0.0774) *p* < 0.001	0.262, (0.0709) *p* < 0.001	0.0771–0.2909	0.0112	40.10%
BAI	0.4664, (0.065) *p* < 0.001	0.3494, (0.0633) *p* < 0.001	0.052–0.1997	0.0085	25.09%

BAI, beck anxiety inventory; BDI, beck depression inventory.

## Discussion

In the present study, by investigating hospitalized NSSI adolescents, we demonstrated that the NSSI adolescents scored higher on childhood abuse than HC adolescents. NSSI adolescents with childhood traumatic experiences have stronger impulsivity, and more severe clinical outcomes than those without, and impulsivity is a significant mediator of the effect of childhood maltreatment on the frequency of self-injury, depression and anxiety symptoms in NSSI adolescents.

Overall, all types of maltreatment experience increased adolescent engagement in NSSI, which is consistent with most studies. However, the processes that act on NSSI may be distinct for different types of abuse histories. Emotional abuse is directly associated with NSSI, whereas the effects of sexual and physical abuse may be indirect (36). From the perspective of stress regulation, this may be associated with activation patterns of the hypothalamic–pituitary–adrenal axis (HPA axis). Emotional abuse may impair the resilience of HPA axis to acute stress response, and repeated exposure to physical abuse may affect the sensitivity of HPA axis (37). The present findings also suggested that adolescents with NSSI showed higher levels of impulsivity, and impulsivity was positively correlated with NSSI frequency and severity of depressive and anxiety symptoms (38–40). A longitudinal study identified impulsivity as a potential risk factor for NSSI, independently predicting the new onset of NSSI (41). This suggests that those who act quickly and without thoughtful consideration are more likely to self-injury than those who think deeply (42).

The prevalence of emotional abuse, physical abuse, sexual abuse, emotional neglect, and physical neglect (47.5, 28.75, 17.5, 70.63, and 56.88%, respectively) among the NSSI adolescents in this study were higher than previously reported global and Chinese adolescent prevalence rates (43, 44). This may be due to the different sample sizes and the higher disease severity in hospitalized patients. However, the prevalence of our sample was consistent with another study of Chinese college students (45), with the highest prevalence of physical neglect and emotional neglect and the lowest prevalence of sexual abuse. Moreover, NSSI adolescents who experience childhood maltreatment report more symptoms of depression and anxiety. Most previous studies have focused on sexual abuse, but a growing body of evidence finds that emotional abuse/neglect exerts an even greater effect on mental health (15). Emotional abuse is often a long-term chronic state that may cause individuals to become trapped in adverse and dysregulated emotions, leading to dysfunctional behaviors of coping (46), such as self-injury and suicide. The present study also found that NSSI adolescents exposed to emotional abuse had more frequency of self-injury.

In addition, NSSI adolescents with a history of childhood maltreatment have higher impulsivity, which is in line with the results of past studies (47, 48). In a longitudinal study of adolescents, a history of physical abuse and physical neglect was found to significantly predict subsequent impulsivity levels (49). Another study found that emotional abuse/neglect was strongly associated with impulsivity (50). And a study of resting-state functional magnetic resonance imaging in adults may explain the neurophysiological mechanism of childhood maltreatment affecting impulsivity (51). The study found that adults with childhood maltreatment experience self-reported higher impulsivity, and reduced functional connectivity (FC) between the inferior parietal lobule and the middle frontal gyrus, superior temporal gyrus and dorsal anterior cingulate cortex. Further mediation analysis found that the effect of childhood maltreatment on impulsivity was mediated by FC between the inferior parietal lobule and the prefrontal cortex. Consequently, childhood maltreatment induces alterations in brain regions associated with response inhibition, which would enhance impulsivity and further increase vulnerability to adverse outcomes (21, 52). This contributes to the understanding of the core findings of this study, where impulsivity partially mediated the effects of childhood maltreatment on depression, anxiety symptoms, and NSSI frequency in adolescents with NSSI. When facing stressful events and experiencing negative emotions, impulsivity may drive individuals toward self-injurious behavior without adequate consideration of the potential negative consequences (53). As time goes on, the frequency and severity of NSSI increases (54). Adolescence is a critical stage of lifespan characterized by substantial neurodevelopment and a spontaneous increase in impulsivity (55). The onset of NSSI was 12–14 years (56), which tends to increase in early adolescence (57), while declining substantially in late adolescence and slowly in early adulthood (58). This characteristic overlaps with the developmental features of brain impulsivity. Traumatic experiences may have long-term neurobiological effects on the human brain resulting in psychopathology (59). Early-life adversity triggers changes in brain structure and function, including emotional and control brain regions. Abnormalities in emotion-related brain areas may lead to emotional dysregulation. Subsequently, dysfunction in control-related brain areas results in impaired inhibitory control and exhibits abnormally high impulsivity (60). Individuals might hardly suppress their negative emotions and self-injurious impulses, then engage in self-injurious behaviors uncontrollably, which may be one of the neurophysiological mechanisms of self-injurious behaviors.

## Limitations

There were several limitations in the current study. At first, the cross-sectional design of this study cannot reveal the causality of childhood maltreatment experiences, trait impulsivity, and relevant characteristics of NSSI behavior. Longitudinal studies were required to help validate the mediating model in NSSI adolescents. Second, We did not include adolescents with psychiatric disorders without NSSI as another control group, and a comparison of adolescents with psychiatric disorders with and without NSSI will improve the convincing of the conclusions. Third, the severity of NSSI was not assessed, which is a complex concept that may require weighting for frequency of NSSI, degree of physical injury of body regions, etc. Future study is needed to improve the comprehensiveness of the NSSI measurement. Besides, the mechanisms and risk factors for NSSI require further investigation, which helps the development of more effective prevention and intervention for NSSI.

## Conclusion

This work highlights the importance of childhood maltreatment and trait impulsivity for NSSI. Our findings further clarify the direct effects and mediating role of childhood maltreatment and impulsivity on NSSI-related clinical outcomes, respectively, broadening the research on NSSI. The results also have implications in NSSI clinical practices that trait impulsivity and childhood maltreatment could be potential targets in NSSI intervention and prevention.

## Data availability statement

The raw data supporting the conclusions of this article will be made available by the authors, without undue reservation.

## Ethics statement

The studies involving human participants were reviewed and approved by the ethics committee from Ningbo Kangning Hospital. Written informed consent to participate in this study was provided by the participants’ legal guardian/next of kin.

## Author contributions

XL, X-LL, and Y-JW had full access to all study data and take responsibility for the integrity of the data and the accuracy of the data analysis, and drafted the manuscript. XL, X-LL, Y-JW, D-SZ, and T-FY were responsible for the study of concept and design, and critically revised the manuscripts. All authors were involved in the acquisition and interpretation of the data, and read and approved the final manuscript.

## Funding

This study was funded by the Medical and Health Brand Discipline in Ningbo (PPXK2018-08).

## Conflict of interest

The authors declare that the research was conducted in the absence of any commercial or financial relationships that could be construed as a potential conflict of interest.

## Publisher’s note

All claims expressed in this article are solely those of the authors and do not necessarily represent those of their affiliated organizations, or those of the publisher, the editors and the reviewers. Any product that may be evaluated in this article, or claim that may be made by its manufacturer, is not guaranteed or endorsed by the publisher.
