# Supplementary material for: The effects of childhood maltreatment on adolescent non-suicidal self-injury behavior: mediating role of impulsivity
Source: Front Psychiatry. 2023 May 26;14:1139705. doi: 10.3389/fpsyt.2023.1139705 (PMC10250706; doi:10.3389/fpsyt.2023.1139705)
Supplement: Supplementary file 1 [file Table_1.docx]

***Supplementary Material***

**Article Title**

**Xi Li ^1, #^, BSc; Xiao-Li Liu ^2, #^, MSc; Yu-Jing Wang ^1, #^, BSc; Dong-Sheng Zhou ^2, *^, MD; Ti-Fei Yuan ^1, 3, 4, *^, PhD**

^1^ School of Mental Health, Wenzhou Medical University, Wenzhou, China

^2^ Department of Psychiatry, Ningbo Kangning Hospital, Ningbo, Zhejiang, China

^3^ Shanghai Key Laboratory of Psychotic disorders, Shanghai Mental Health Center, Shanghai Jiao Tong University School of Medicine, Shanghai, China

^4^ Co-innovation Center of Neuroregeneration, Nantong University, Nantong, China

# LX, XLL and YJW contributed equally to this work.

* Correspondence: Corresponding Author: ytf0707@126.com (TFY), wyzhouds@sina.com (DSZ)

**Supplementary Table 1.** Group differences in clinical characteristics of NSSI adolescents with and without emotional abuse.

|  | Emotional abuse | | Statistic (χ²/*z*) | *p* |
| --- | --- | --- | --- | --- |
|  | Without(N=84) | With(N=76) |  |  |
| Age, year | 14.27(1.426) | 14.04(1.361) | 1.061 | 0.29 |
| Sex (M, F) | 18,66 | 8,68 | 3.485 | 0.062 |
| NSSI frequency score  (past month) | 1.6(0.995) | 1.93(0.869) | -2.299 | 0.023 |
| BAI | 18.87(12.097) | 30.78(12.948) | -6.013 | < 0.001 |
| BDI | 25.05(15.386) | 37.46(13.165) | -5.455 | < 0.001 |
| BIS | 52.2913(15.983) | 61.3595(15.624) | -3.622 | < 0.001 |

BAI, Beck Anxiety Inventory; BDI, Beck Depression Inventory; BIS, Barratt Impulsiveness Scale.

**Supplementary Table 2.** Group differences in clinical characteristics of NSSI adolescents with and without physical abuse

|  | Physical abuse | | Statistic (χ²/*z*) | *p* |
| --- | --- | --- | --- | --- |
|  | Without (N = 114) | With (N = 46) |  |  |
| Age, year | 14.26(1.488) | 13.91(1.112) | 1.627 | 0.107 |
| Sex (M, F) | 18,96 | 8,38 | 0.062 | 0.804 |
| NSSI frequency score  (past month) | 1.69(0.97) | 1.91(0.89) | -1.329 | 0.186 |
| BAI | 22.65(13.348) | 29.17(14.021) | -2.758 | 0.006 |
| BDI | 29.7(15.813) | 34.02(14.853) | -1.591 | 0.114 |
| BIS | 55.1604(15.900) | 60.1633(17.259) | -1.757 | 0.081 |

BAI, Beck Anxiety Inventory; BDI, Beck Depression Inventory; BIS, Barratt Impulsiveness Scale.

**Supplementary Table 3.** Group differences in clinical characteristics of NSSI adolescents with and without sexual abuse.

|  | Sexual abuse | | Statistic (χ²/*z*) | *p* |
| --- | --- | --- | --- | --- |
|  | Without (N = 132) | With(N=28) |  |  |
| Age, year | 14.17(1.316) | 14.11(1.75) | 0.23 | 0.818 |
| Sex (M, F) | 24,108 | 2,26 | 0.257 | 0.12 |
| NSSI frequency score  (past month) | 1.72(0.952) | 1.93(0.94) | -1.057 | 0.292 |
| BAI | 23.11(13.309) | 31.21(14.472) | -2.884 | 0.004 |
| BDI | 29.31(15.335) | 38.64(14.88) | -2.94 | 0.004 |
| BIS | 55.5173(16.657) | 61.6964(14.354) | -1.823 | 0.07 |

BAI, Beck Anxiety Inventory; BDI, Beck Depression Inventory; BIS, Barratt Impulsiveness Scale.

**Supplementary Table 4.** Group differences in clinical characteristics of NSSI adolescents with and without emotional neglect.

|  | Emotional neglect | | Statistic (χ²/*z*) | *p* |
| --- | --- | --- | --- | --- |
|  | Without (N = 47) | With (N = 113) |  |  |
| Age, year | 14.38(1.568) | 14.07(1.314) | 1.201 | 0.234 |
| Sex (M, F) | 8,39 | 18,95 | 0.029 | 0.865 |
| NSSI frequency score  (past month) | 1.7(0.976) | 1.78(0.942) | -0.464 | 0.644 |
| BAI | 18.11(12.452) | 27.19(13.53) | -3.959 | < 0.001 |
| BDI | 24.57(16.663) | 33.59(14.431) | -3.438 | < 0.001 |
| BIS | 48.7409(15.994) | 59.867(15.502) | -4.097 | < 0.001 |

BAI, Beck Anxiety Inventory; BDI, Beck Depression Inventory; BIS, Barratt Impulsiveness Scale.

**Supplementary Table 5.** Group differences in clinical characteristics of NSSI adolescents with and without physical neglect.

|  | Physical neglect | | Statistic (χ²/*z*) | *p* |
| --- | --- | --- | --- | --- |
|  | Without (N = 69) | With (N = 91) |  |  |
| Age, year | 14.26(1.462) | 14.09(1.347) | 0.775 | 0.439 |
| Sex (M, F) | 13,56 | 13,78 | 0.598 | 0.439 |
| NSSI frequency score  (past month) | 1.83(0.969) | 1.7(0.937) | 0.809 | 0.42 |
| BAI | 20.22(12.777) | 27.79(13.753) | -3.556 | < 0.001 |
| BDI | 26.06(16.233) | 34.65(14.135) | -3.57 | < 0.001 |
| BIS | 51.2562(17.293) | 60.6496(14.527) | -3.73 | < 0.001 |

BAI, Beck Anxiety Inventory; BDI, Beck Depression Inventory; BIS, Barratt Impulsiveness Scale

**Supplementary Table 6.** The relationship between impulsivity, childhood maltreatment, and clinical outcome in NSSI group.

|  |  | Emotion abuse | Physical abuse | Sexual abuse | Emotional neglect | Physical neglect | CTQ total | BIS total |
| --- | --- | --- | --- | --- | --- | --- | --- | --- |
| NSSI frequency score  (past month) | r | 0.245 | 0.027 | 0.073 | 0.033 | 0.027 | 0.155 | 0.319 |
|  | *p* | 0.002 | 0.732 | 0.36 | 0.68 | 0.735 | 0.051 | <0.001 |
| BAI | r | 0.517 | 0.190 | 0.254 | 0.252 | 0.332 | 0.492 | 0.504 |
|  | *p* | <0.001 | 0.016 | 0.001 | 0.001 | <0.001 | <0.001 | <0.001 |
| BDI | r | 0.447 | 0.082 | 0.228 | 0.262 | 0.315 | 0.423 | 0.590 |
|  | *p* | <0.001 | 0.305 | 0.004 | <0.001 | <0.001 | <0.001 | <0.001 |

BAI, Beck Anxiety Inventory; BDI, Beck Depression Inventory; BIS, Barratt Impulsiveness Scale; CTQ, Childhood Trauma Questionnaire.
